# Application of a Combined Transcriptomics and Proteomics Analysis Approach to Explore Potential Mechanisms of Biomarkers in NIHL

**DOI:** 10.1002/cns.70935

**Published:** 2026-08-27

**Authors:** Zhong‐Jia Ding, Chao‐Yong Tian, Ren‐Feng Wang, Yin Wang, Ding‐Jun Zha

**Affiliations:** ^1^ Department of Otolaryngology Xijing Hospital, Air Force Military Medical University Xi'an Shaanxi China; ^2^ Department of Ultrasound Medicine Xi'an Daxing Hospital Xi'an Shaanxi China; ^3^ Yan'an University Yan'an China

**Keywords:** biomarker, cochlear inflammation, integrated transcriptomics and proteomics, mitochondrial energy metabolism, noise‐induced hearing loss, translocator protein

## Abstract

**Objective:**

This study aimed to elucidate the molecular mechanisms underlying noise‐induced hearing loss (NIHL) by analyzing cochlear protein expression and transcriptional profiles at multiple time points after noise exposure. This investigation aimed to identify key biomarkers and potential therapeutic targets to mitigate the irreversible effects of NIHL.

**Methods:**

Cochlear specimens were collected from animal models at 1, 14, and 28 days post‐noise exposure. Integrated transcriptomic and proteomic analyses were performed to identify differentially expressed genes and proteins, followed by bioinformatic analyses to explore their functional roles. Reverse transcription quantitative PCR (RT‐qPCR) was used to validate the expression levels of selected genes.

**Results:**

Six biomarkers (Gm37065, Cd5l, F930017D23Rik, Eldr, Gm30948, and Tspo2) showed strong correlations with NIHL. RT‐qPCR validation revealed a significant decrease in mRNA expression of five of these genes, including Gm37065, across all experimental groups, while the expression of Cd5l remained unchanged. Functional analyses indicated that disruptions in mitochondrial energy metabolism and cochlear inflammation are key contributors to NIHL pathogenesis.

**Conclusion:**

This study reveals dynamic molecular changes associated with NIHL progression and identifies novel biomarkers and pathways with potential therapeutic relevance. Further mechanistic validation and functional studies are necessary to elucidate the roles of Tspo, Cd5l, and other identified molecules in NIHL pathogenesis and to develop targeted therapeutic strategies.

## Graphical abstract

This study performed a time‐series analysis of cochlear tissues from mice prior to noise exposure (CN) and at 1, 14, and 28 days post‐exposure. Through the integration of transcriptomics and proteomics techniques, we identified dynamically differentially expressed genes (DEGs) and proteins (DEPs). Correlation analysis further identified six biomarkers significantly associated with noise‐induced hearing loss (NIHL): Gm37065, Cd5l, F930017D23Rik, Eldr, Gm30948, and Tspo2. To elucidate the functions and mechanisms of these biomarkers, we employed bioinformatics approaches such as gene set enrichment analysis (GSEA), gene–gene interaction (GGI) network construction, and regulatory network analysis involving transcription factors (TFs), mRNAs, long non‐coding RNAs (lncRNAs), and microRNAs (miRNAs). Additionally, reverse transcription quantitative PCR (RT‐qPCR) was used to validate the expression levels of the selected biomarkers, confirming their association with NIHL and revealing potential regulatory pathways. These findings offer a comprehensive understanding of cochlear pathological changes induced by NIHL and lay a solid foundation for further investigation into its molecular mechanisms. This research provides valuable insights into the development of diagnostic markers and therapeutic targets for NIHL.

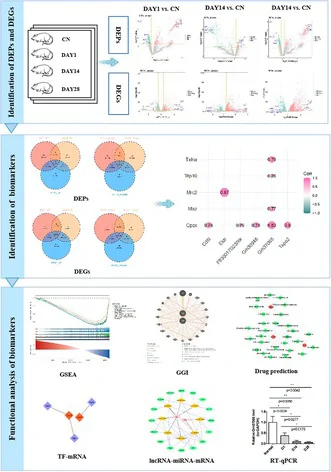

## 1 Introduction

Noise‐induced hearing loss (NIHL) is hearing loss caused by long‐term exposure to high‐intensity noise, resulting in damage to the auditory system and leading to the death of cochlear hair cells and degeneration of spiral ganglion neuron ribbon synapses, causing irreversible damage. NIHL accounts for one‐third of global hearing impairments and is a major cause of occupational disability worldwide, posing significant social and physiological challenges [1]. The mechanisms underlying NIHL are complex, with oxidative damage and mechanical noise injury being key contributors [2; 3]. Therapeutic targets for hearing restoration are still being explored. Although reliance on hearing aids and cochlear implants can improve hearing, they are unable to fundamentally repair damaged hair cells and restore spiral ganglion neuron function. Therefore, a deeper understanding of the mechanisms of NIHL is crucial for identifying potential repair targets.

Proteomics, the large‐scale study of proteins, reveals the expression levels, structural characteristics, functional roles, interactions, and posttranslational modifications of proteins within cells or organisms [4]. It is invaluable in disease research and has been widely used in recent years to explore differential proteins in diseases such as diabetes and Alzheimer's disease [5]. Comparative proteomics based on mass spectrometry can screen for key protein changes on a larger scale, and multiomics integrated analysis offers advantages in uncovering disease mechanisms [6]. For example, subtle patterns in gene expression data can be enhanced through epigenomics. Recent studies have shown that combining transcriptomics and proteomics data can help identify pathogenic genes associated with migraine and therapeutic targets for chronic kidney disease [7]. However, key targets for noise‐induced and drug‐induced hearing loss remain understudied.

In this study, 1 day, 14 days, and 28 days after noise exposure were selected as the time points for cochlear damage. By analyzing transcriptomic and proteomic sequencing data, we aimed to identify changes in cochlear protein expression and function during the acute and chronic phases of NIHL, explore key biomarkers associated with NIHL, and investigate mechanisms related to the irreversibility of NIHL. This research provides new directions for the prevention and treatment of NIHL.

## 2 Materials and Methods

### 2.1 Laboratory Animals and Sample Collection

The research protocol was approved by the Fourth Military Medical University Ethics Committee. This study employed 12 healthy 8‐week‐old Sprague–Dawley (SD) rats (weighing 100–200 g) that were fed ad libitum and exhibited sensitive pinna reflexes, with no history of exposure to noise, ototoxic drugs, or otitis media. Twelve SD rats were randomly divided into four groups, with three replicates in each group: a blank control group (CN), a noise exposure 1 day group (Day 1), a noise exposure 14 day group (Day 14), and a noise exposure 28 day group (Day 28). Rats in the experimental group were placed in a soundproof chamber (including details such as instrument model, specifications, and origin) and exposed to 8–16 kHz continuous narrowband noise at an intensity of 110 dB SPL for 2 h. Rats in the blank control group were placed in the same soundproof chamber under identical environmental conditions (temperature: 22°C ± 2°C, humidity: 50% ± 5%) without noise exposure. A sound level meter (Bruel and Kjaer, Type 2606) was positioned at the level of the rats' ears to monitor noise intensity and frequency in real time. At the end of the experiment, cochlear samples from each group of rats were collected for proteomic and transcriptomic sequencing.

### 2.2 Data Preprocessing

The raw proteomic data were preprocessed via the R package MaxQuant (version 1.6.4) [8], with TMT‐9plex set as the quantitative method. The false discovery rate for protein and peptide identification was adjusted to be less than 1%, and the minimum score for peptides was set to be greater than 40.

The raw transcriptome data were filtered and quality controlled via the R package Trimmomatic (version 0.39) [9], which was employed to remove junctions in the Fastq sequences from the Illumina platform and trim Fastq sequences on the basis of base quality values. Following filtering, the R package HISAT2 (version 2.2.1) [10] was used to compare the reads with the reference genes of the rats. The R package SAMtools (version 1.16.1) [11] was subsequently used to count the comparisons, with reads mapping primarily used to test the overall alignment rate. The R package feature Counts (version 2.0.3) [12] was then employed to quantify the previously compared reads on the basis of the reference genome annotation file. The RNA count value obtained from featureCounts was used for differential expression analysis.

### 2.3 Differential Expression Analysis

For the proteomic data, differential expression analyses of differentially expressed proteins (DEPs) 1 (Day 1 vs. CN), DEPs2 (Day 14 vs. CN), and DEPs3 (Day 28 vs. CN) were performed via the R package limma (version 3.56.2) [13] (|log_2_FoldChange(FC)| ≥ 0.5, *p* ≤ 0.05). Similarly, in the transcriptome data, differential expression analyses of differentially expressed genes (DEGs) 1 (Day 1 vs. CN), DEGs 2 (Day 14 vs. CN), and DEGs 3 (Day 28 vs. CN) were performed via the R package DESeq2 (version 1.40.2) [14] (|log_2_FC| ≥ 1, adj. *p* ≤ 0.05). Volcano and heatmaps of DEPs1, DEPs2, and DEPs3 and DEGs1, DEGs2, and DEGs3 were then generated via the R packages ggplot2 (version 3.4.4) [15], ComplexHeatmap (version 2.16.0) [16] and circlize (version 0.4.15) [17], respectively, to visualize their expression.

### 2.4 Functional Annotation of DEPs and DEGs

To elucidate the potential biological functions and pathways of the DEPs and DEGs, Gene Ontology (GO) (adj. *p* < 0.05), and Kyoto Encyclopedia of Genomes (KEGG) enrichment analyses (*p* < 0.05) were performed on DEPs1, DEPs2, and DEPs3 via the R packages clusterProfiler (version 4.8.2) [18] and org.Mm.eg.db (version 3.16.0) [19]. Similarly, GO and KEGG enrichment analyses (adj. *p* < 0.05) of DEGs1, DEGs2, and DEGs3 were performed via the R packages clusterProfiler (version 4.8.2) [18] and org.Mm.eg.db (version 3.16.0) [19]. The GO analysis covered three main categories: biological process (BP), cellular component (CC), and molecular function (MF).

### 2.5 Consensus Biomarkers and Pathways Identified by Integrated Analysis

The R package gegan (version 0.1.10) [20] was used to perform Venn analysis on the differentially upregulated and downregulated proteins, resulting in the identification of candidate DEPs. To identify genes consistently up/downregulated with time (CONDEGs) following NIHL, a temporal clustering approach, which facilitates multiple assignments of a specific gene across different clusters on the basis of the representation of DEGs within those clusters, was applied to the DEGs observed in the CN and NIHL groups at various time points. This analysis was conducted via the Mfuzz package (version 2.58.0) [21]. Specifically, the number of clusters was predetermined to be 10, and a threshold score of 0.9 was established to determine the clusters with which each DEG was affiliated. Venn analysis was performed via gegan (version 0.1.10) [20] to obtain CONDEGs following NIHL. Molecules with consistent expression trends (e.g., DEPs and DEGs that are upregulated/downregulated simultaneously) are defined as candidate consensus biomarkers. Correlation analysis (Spearman) was performed on the candidate DEPs and CONDEGs, with the threshold set at a correlation coefficient (Cor) > 0.75 and *p* < 0.05, to obtain the biomarkers and draw a correlation heatmap.

### 2.6 Gene Set Enrichment Analysis (GSEA)

To further evaluate the signaling pathways associated with the biomarkers, GSEA was performed via the R package clusterProfiler (version 4.8.2) [18]. The KEGG reference gene set was “m2.cp.v2023.2.Mm.entrez.gmt,” which contains 186 gene sets. Thresholds were set at adj. *p* < 0.05 and |normalized enrichment score (NES)| > 1.

### 2.7 GeneMANIA Analysis

To explore the interactions between biomarkers and other genes in detail, coexpression networks were mapped via GeneMANIA (http://www.genemania.org/) to reveal the internal connections between genes.

### 2.8 Analysis of Molecular Regulatory Networks and Drug Prediction

To obtain the molecular regulatory network of the biomarkers, relevant microRNAs (miRNAs) were predicted via NetworkAnalyst (https://www.networkanalyst.ca/), and relevant lncRNAs were predicted via the predicted miRNAs in Encori (https://ngdc.cncb.ac.cn/) to obtain the miRNA–long noncoding RNA (lncRNA) pairs. Transcription factors (TFs) were further predicted via the NetworkAnalyst platform (https://www.networkanalyst.ca/), and the mRNA–miRNA‐lncRNA and mRNA‐TF networks were visualized via Cytoscape software (version 3.7.1) [22]. To explore potential drugs for the treatment of NIHL patients, drug prediction on the basis of biomarkers was performed via the Comparative Toxicogenomics Database (CTD) (https://ctdbase.org/), and then, biomarker–drug networks were visualized via Cytoscape software (version 3.7.1) [22].

### 2.9 Reverse Transcription–Quantitative Polymerase Chain Reaction (RT–qPCR)

RNA was extracted from 12 frozen cochlear tissue samples via a TRIzol kit. The experimental design featured groups 1–3 as controls, with groups 4–6 exposed to noise for 1 day, groups 7–9 for 14 days, and groups 10–12 for 28 days. All experimental steps for total RNA extraction were performed according to the instructions. One microliter of extracted RNA was taken for concentration detection with a NanoPhotometer N50, and the purity/concentration was recorded to calculate the amount of RNA for subsequent reverse transcription steps. The RNA was subsequently reverse transcribed into cDNA via Servicebio's SweScript First Strand cDNA Synthesis Kit according to the instructions. Next, the cDNA was diluted 5–20 times with ddH2O (without RNase/ARase), and 3 μL of cDNA, 5 μL of 2 × Universal Blue SYBR Green qPCR Master Mix, 1 μL of forward primer (10 μM) and 1 μL of reverse primer (10 μM) were added. In addition, 40 cycles of reactions were performed via the CFX96 real‐time quantitative PCR instrument, and the program information is provided in Table S1. Primer sequence information for Gm37065, Cd5l, F930017D23Rik, Eldr, Gm30948, and Tspo2 is shown in Table S2. GAPDH was used as a reference gene, and relative gene expression levels were determined via the 2^−△△CT^ method.

### 2.10 Statistical Analysis

All the statistical analyses were performed via the R software (version 4.2.2), with significant differences between groups assessed via the Wilcoxon test (*p* < 0.05).

## 3 Results

### 3.1 Identification of DEPs and DEGs

**FIGURE 1:**
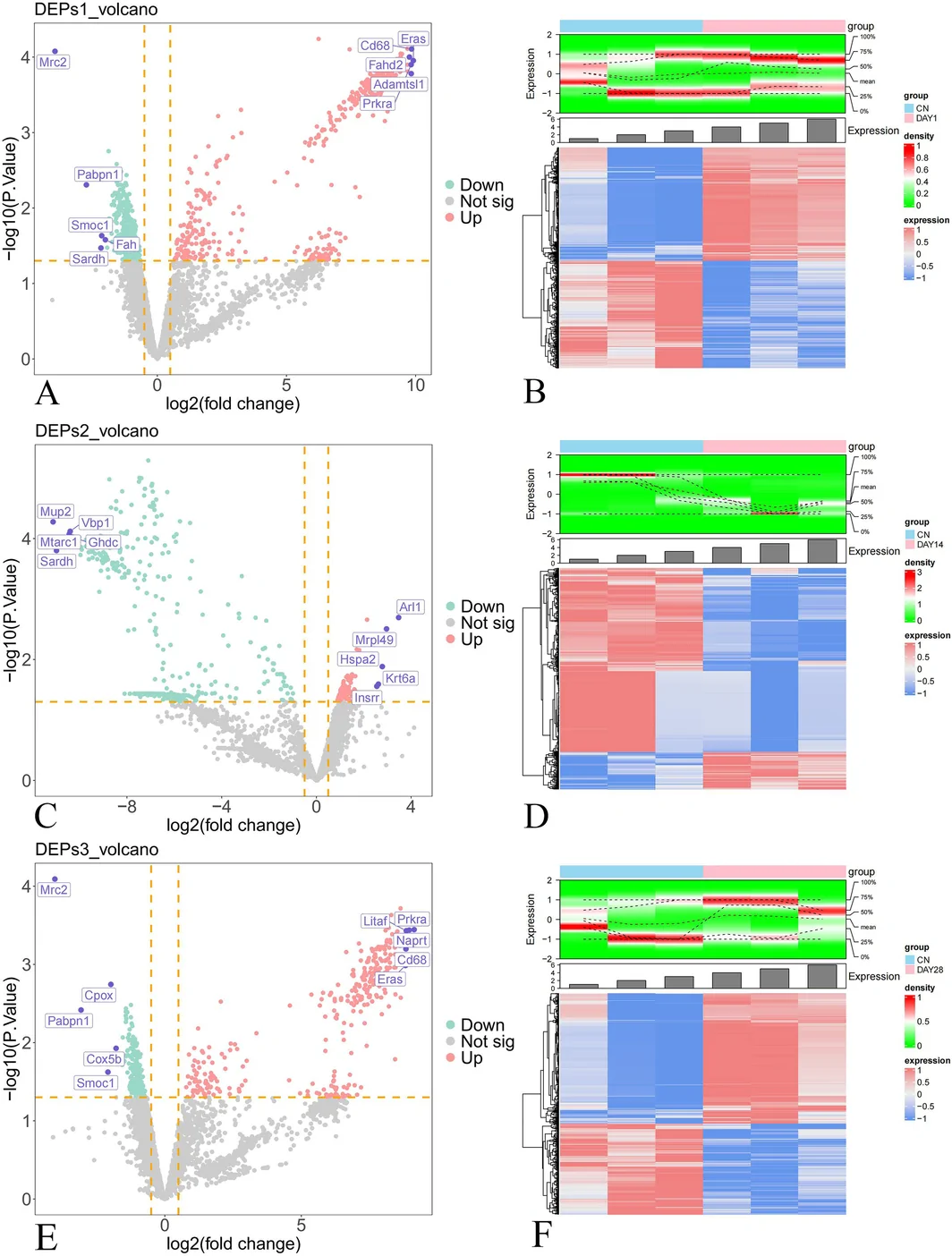
Volcano and heatmap of all differentially expressed proteins between different groups. (A, B) represent the volcano and heatmap of differential proteins between Day 1 versus CN. (C, D) represent the volcano and heatmap of differential proteins between Day 14 versus CN. (E, F) represent the volcano and heatmap of differential proteins between Day 28 versus CN. Different colored dots represent differentially expressed proteins with increased/decreased/no statistical significance.

The results of protein differential expression analysis revealed a total of 3457 proteins, of which DEP1 (Day 1 vs. CN) contained 374 downregulated proteins and 395 upregulated proteins (Figure 1A,B), DEP2 (Day 14 vs. CN) contained 384 downregulated proteins and 77 upregulated proteins (Figure 1C,D), and DEP3 (Day 28 vs. CN) contained 204 downregulated proteins and 294 upregulated proteins (Figure 1E,F). Further analysis of the DEGs revealed that DEGs1 (Day 1 vs. CN) included 136 downregulated genes and 201 upregulated genes (Figure S1), that DEGs2 (Day 14 vs. CN) included 296 downregulated genes and 730 upregulated genes (Figure S1), and that DEGs3 (Day 28 vs. CN) included 394 downregulated genes and 1108 upregulated genes (Figure S1).

### 3.2 Enrichment Analysis of DEPs

**FIGURE 2:**
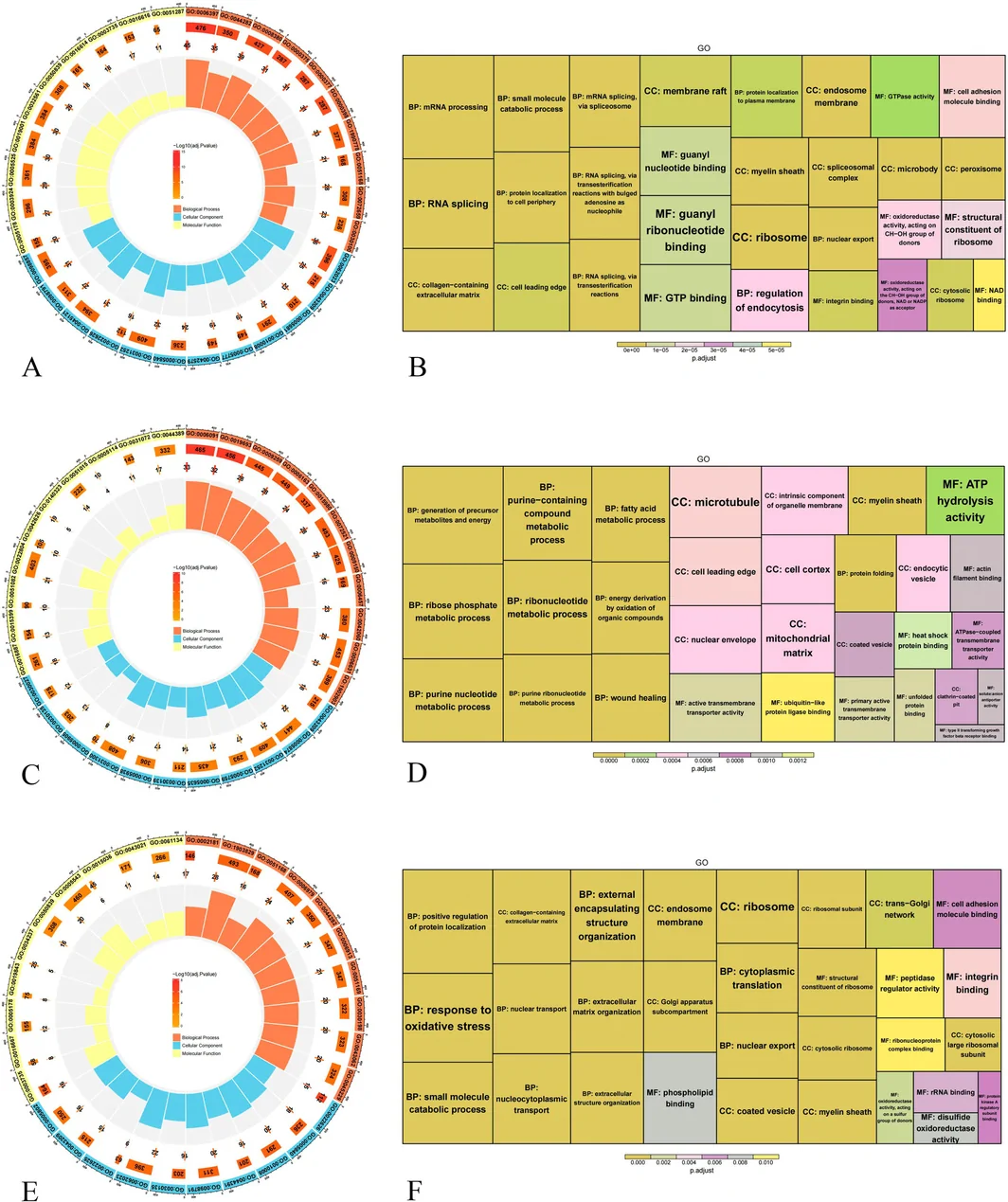
Pathway enrichment analysis of differentially expressed proteins between different groups based on the Go database. (A, B) represent the enrichment analysis circle and block diagram of the differential protein go database pathway between Day 1 versus CN. (C, D) represent the differential protein GO database pathway enrichment analysis circle and block diagrams of Day 14 versus CN. (E, F) represent the differential protein GO database pathway enrichment analysis circle and block diagrams for Day 28 versus CN. The different colors in the circle chart represent different types of pathways, and the different circles represent the relative number of proteins enriched in the pathways. The different colors in the block diagram represent different *p*‐values, and the larger the block area of the pathway, the more proteins are enriched.

When the GO enrichment results were analyzed, the BPs associated with DEP1 showed a predominance of messenger RNA (mRNA) processing and small molecule catabolic processes. Additionally, the CCs were primarily concentrated within the membrane raft and endosome membrane, and the MFs were enriched mainly in integrin binding and guanylribonucleotide binding (Figure 2A,B). Similarly, the BP analysis of DEPs2 revealed their key roles in the generation of precursor metabolites, energy, and ribose phosphate metabolic processes. The CCs were represented mainly by the myelin sheath and microtubules, and the MFs were involved mainly in ATP hydrolysis activity and heat shock protein binding (Figure 2C,D). Furthermore, DEP3 BPs were enriched in response to oxidative stress and small molecule catabolic processes, CCs were enriched mainly in the endosome membrane and ribosome, and MFs were enriched mainly in the structural constitution of ribosomes and oxidoreductase activity acting on a sulfur group of donors (Figure 2E,F).

The KEGG enrichment results revealed that DEP1 exhibited prominent enrichment in pathways related to amyotrophic lateral sclerosis, Huntington disease and endocytosis (Figure S2A,B). Furthermore, DEPs2 predominantly participated in amyotrophic lateral sclerosis, diabetic cardiomyopathy and the phagosome (Figure S2C,D). DEPs3 was enriched in amyotrophic lateral sclerosis, coronavirus disease‐COVID‐19, and Parkinson's disease (Figure S2E,F).

### 3.3 Enrichment Analysis of DEGs

GO enrichment analysis of the DEGs revealed a predominant enrichment of BPs associated with immune functions, such as B‐cell activation and activation of the immune response. CCs are associated primarily with the immunoglobulin complex and the immunoglobulin complex, which circulate. MFs were enriched mainly in immune receptor activity and immunoglobulin receptor binding (Figure S3A,B). Furthermore, the GO enrichment results of DEG2 revealed that BPs were enriched mainly in the immune response‐regulating cell surface receptor signaling pathway and immune response‐activating signal transduction. CCs are involved mainly in the immunoglobulin complex, the circulation, and the immunoglobulin complex, and MFs are involved mainly in immunoglobulin receptor binding and immune receptor activity (Figure S3C,D). In addition, GO enrichment analysis of DEGs3 revealed a distinct pattern, with BPs enriched primarily in the immune response‐regulating cell surface receptor signaling pathway and immune response‐activating signal transduction. CC enrichment once again underscores the importance of the circulation of immunoglobulin complexes, such as the immunoglobulin complex and the immunoglobulin complex. MFs were enriched mainly in immunoglobulin receptor binding and immune receptor activity (Figure S3E,F). KEGG analysis revealed that DEGs1, DEGs2 and DEGs3 were enriched in 18, 48 and 45 KEGG pathways, respectively (Figure S4). Notably, DEGs1, DEGs2 and DEGs3 were enriched in hematopoietic cell lineage, malaria and cytokine–cytokine receptor interactions.

### 3.4 Acquisition of Six Biomarkers

**FIGURE 3:**
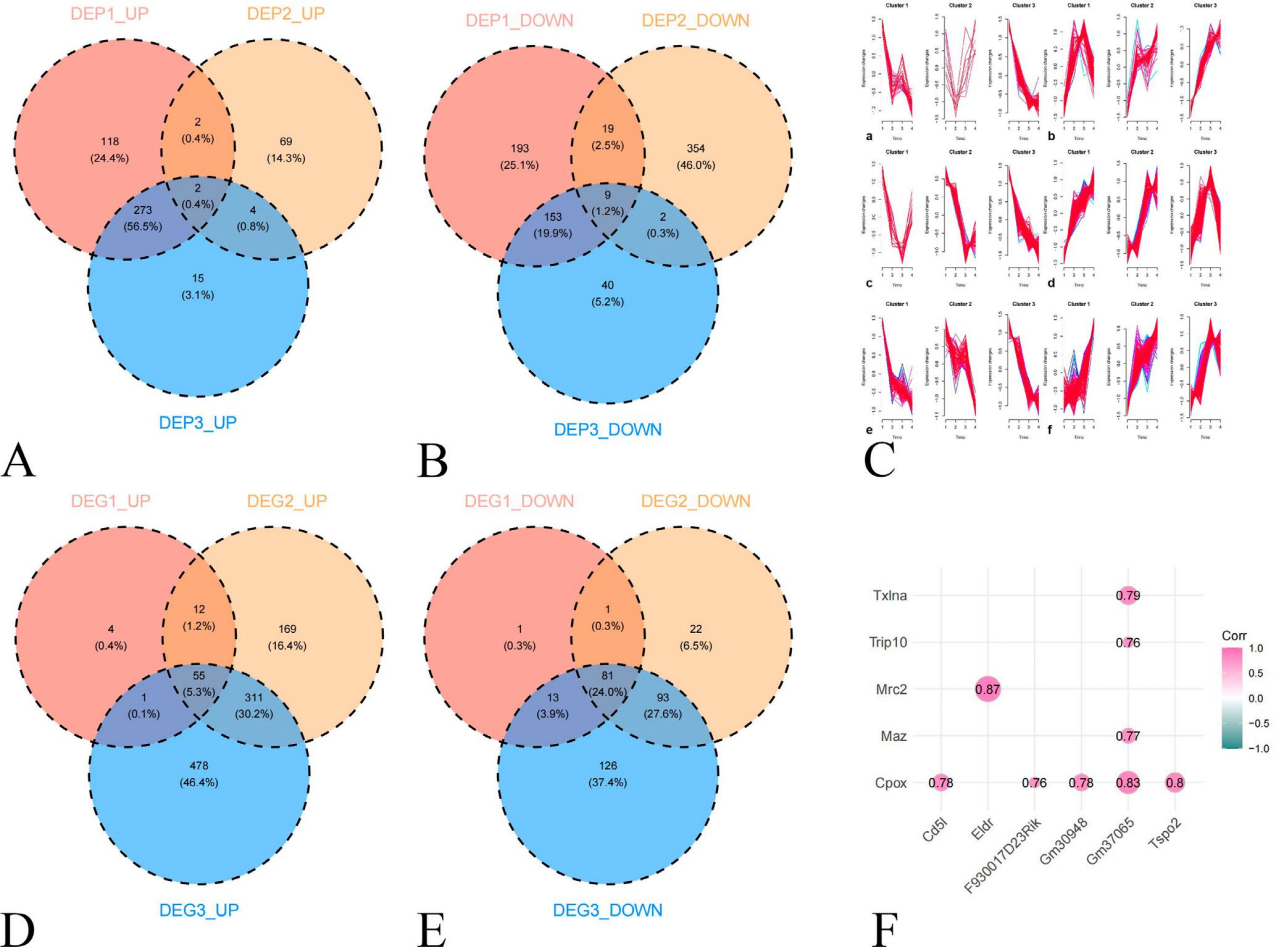
Correlation analysis between differential proteins and differential genes. (A) Venn diagram of three groups of differentially upregulated proteins; (B) venn diagram of three groups of differentially downregulated proteins; (C) time series analysis of differentially expressed genes; (D) venn diagram of three differentially upregulated genes; (E) venn diagram of differentially upregulated genes in three groups; (F) heat map of the correlation between differential proteins and differential genes.

The intersection of the proteins that were both up‐ and downregulated in DEPs1, DEPs2, and DEPs3 was taken to obtain two differentially upregulated proteins (Figure 3A) and nine downregulated proteins (Figure 3B), and these 11 proteins were labeled candidate DEPs, namely, Usp8, Ifit3, Mrc2, Cpox, Vbp1, Slc37a2, Smc4, Trip10, Echdc2, Maz, and Txln. As shown in Figure 3C, the genes whose expression was continuously upregulated/downregulated over time were analyzed. A total of 55 consistently upregulated genes (Figure 3D) and 81 consistently downregulated genes (Figure 3E) were identified, resulting in 136 CONDEGs. Correlation analysis of the DEPs and CONDEGs revealed that five proteins (Cpox, Trip10, Maz, Txlna, and Mrc2) were significantly correlated with six genes (Gm37065, Cd5l, F930017D23Rik, Eldr, Gm30948, and Tspo2), which were defined as biomarkers for further analysis (Figure 3F, Table S4).

### 3.5 GSEA And GeneMANIA Analyses of Six Biomarkers

**FIGURE 4:**
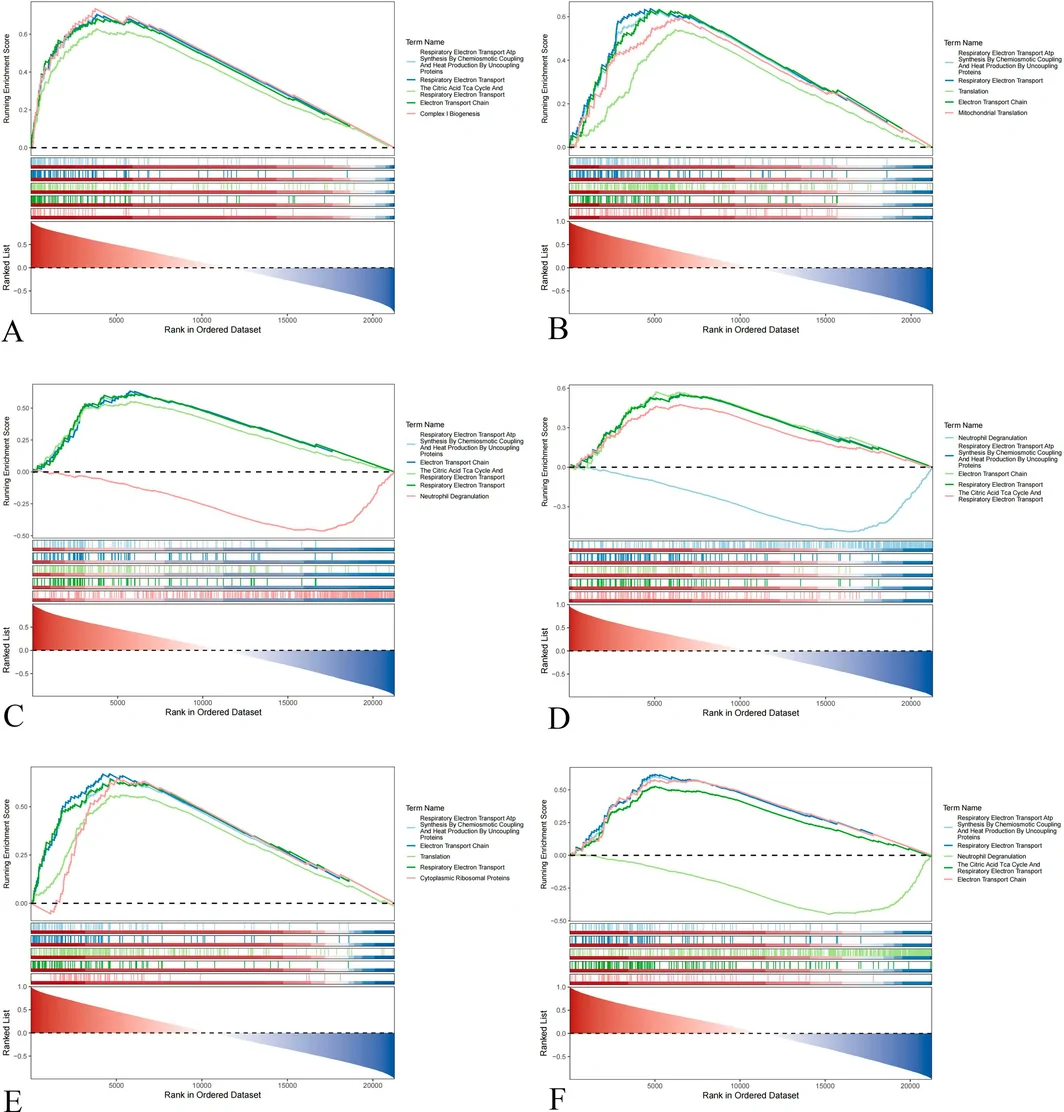
GSEA analysis of key genes in Day 1 versus CN. (A–F) represent the GSEA enrichment of Cd5l, Eldr, F930017D23Rik, Gm30948, Gm37065, and Tspo2, respectively.

GSEA of CNs at different time points (Day 1, Day 14, and Day 28) revealed different enrichment patterns of six biomarkers (Cd5l, Eldr, F930017D23Rik, Gm30948, Gm37065 and Tspo2). At Day 1, these biomarkers are centrally involved in the synthesis and uncoupling of ATP during the respiratory electron transfer of reactants (Figure 4). At Day 14, biomarkers were enriched in the initial triggering of the reaction involving phospholipids and complement (Figure S5), and at Day 28, all of these genes were significantly enriched in the role of phospholipids in the phagocytic response (Figure S6).

### 3.6 Molecular Regulatory Networks of the Six Biomarkers

**FIGURE 5:**
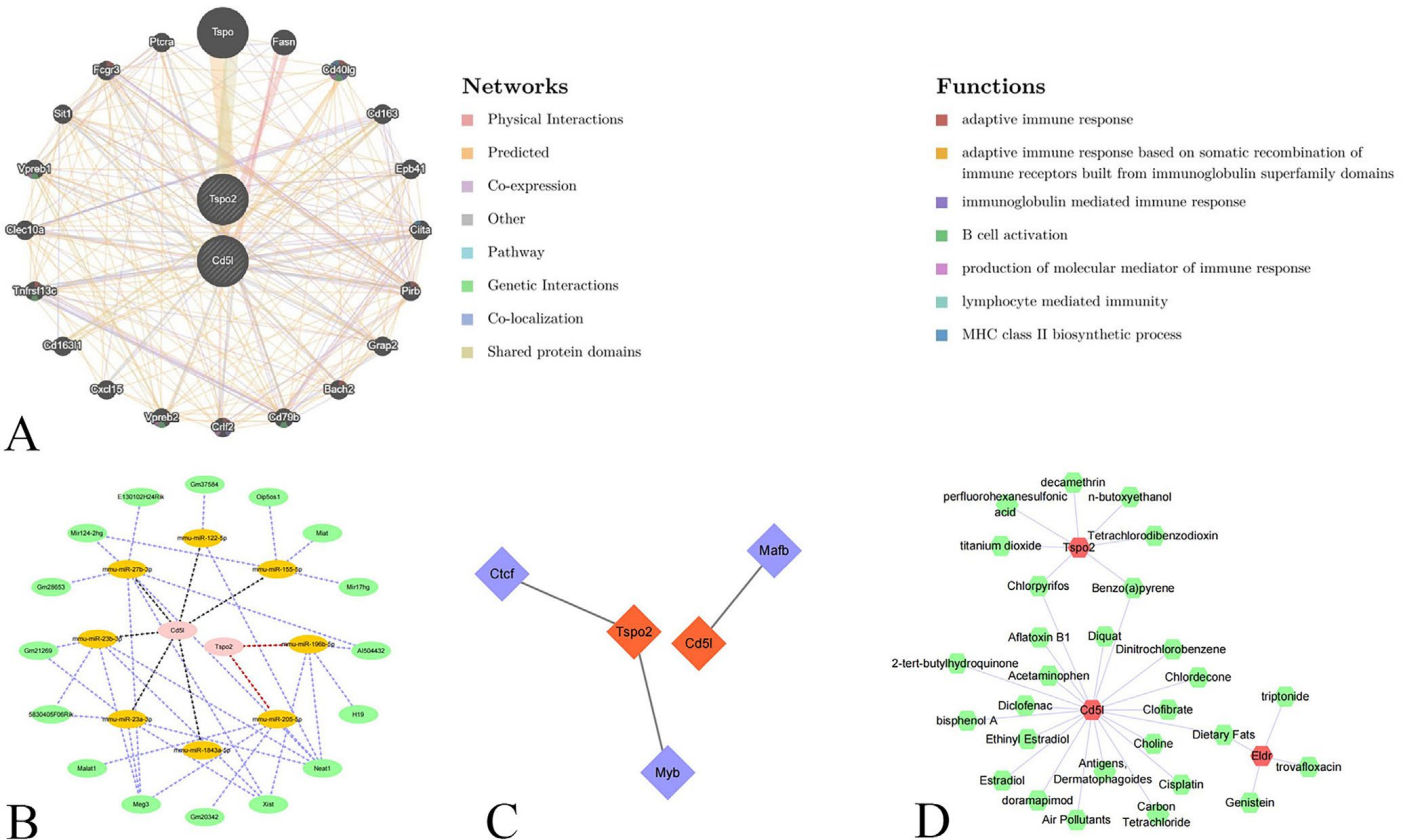
Analysis of the interaction between Tspo2 and Cd5l gene encoded proteins and related proteins. (A) GeneMANIA co‐expression network of Tspo2 and Cd5l. (B) network diagram of mRNA miRNA lncRNA relationship between Tspo2 and Cd5l. (C) network diagram of mRNA TF relationship between Tspo2 and Cd5l. (D) Gene‐drug relationship network diagram of Tspo2 and Cd5l.

GeneMANIA revealed that Tspo2 and Cd5l were associated with genes such as Tspo, Fasn, and Ptcra. The inferred functions primarily involve the adaptive immune response, the adaptive immune response stemming from the somatic recombination of immunoglobulin superfamily domain immune receptors, and B‐cell activation (Figure 5A, Table 1). Among the biomarkers, only Tspo2 and Cd5l were able to predict relevant miRNAs and TFs. Specifically, Tspo2 predicted 2 miRNAs (miR‐196b‐5p and miR‐205‐5p), whereas Cd5l predicted 6 miRNAs, such as miR‐27b‐3p, miR‐122b‐5p, and miR‐155b‐5p, and 32 pairs of miRNA–lncRNA correspondences, such as Mir124‐2hg (Figure 5B). Additionally, Tspo2 predicted 2 TFs (Ctcf, Myb), whereas Cd5l predicted 1 TF (Mafb) (Figure 5C). A total of 28 drugs were predicted by Tspo2, Cd5l and Eldr among the six biomarkers, of which chlorpyrifos and benzo(a)pyrene were predicted by Tspo2 and Cd5l together, and dietary fats were predicted by both Cd5l and Eldr (Figure 5D).

**TABLE 1:** GeneMANIA analysis of key gene.

Function	FDR	Genes in network	Genes in genome
Adaptive immune response	0.004225901	6	294
Adaptive immune response based on somatic recombination of immune receptors built from immunoglobulin superfamily domains	0.004225901	6	281
B cell activation	0.041066211	5	261
Immunoglobulin mediated immune response	0.387653587	3	93
Production of molecular mediator of immune response	0.387653587	4	232
Lymphocyte mediated immunity	0.387653587	4	267
MHC class II biosynthetic process	0.387653587	2	17
Regulation of MHC class II biosynthetic process	0.387653587	2	17
B cell mediated immunity	0.426293812	3	108
Immunoglobulin secretion	0.538773816	2	23

### 3.7 Validation of Six Biomarkers via RT–qPCR

**FIGURE 6:**
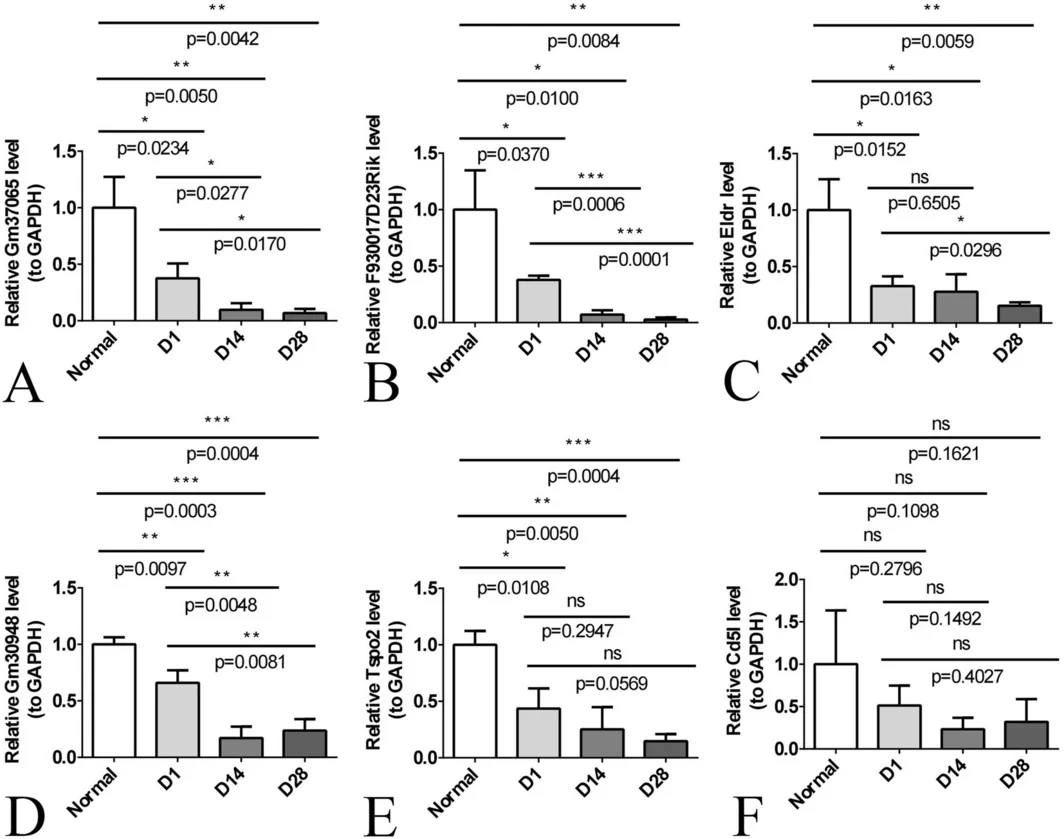
qPCR analysis of key genes between different groups. (A) represents the comparison of expression differences of Gm37065 among different groups. (B) represents a comparison of the expression differences of F930017D23Rik among different groups. (C) represents a comparison of the expression differences of Eldr between different groups; D represents the comparison of expression differences of Gm30948 among different groups. (E) represents a comparison of the expression differences of Tspo2 between different groups. (F) represents the comparison of expression differences of Cd5l among different groups.

The results of the RNA concentration assay revealed that all the samples were within the standard range of RNA concentrations (Table S3). RT–qPCR was then performed on all the samples. RT–qPCR analysis revealed a statistically significant decrease in the mRNA expression levels of Gm37065, F930017D23Rik, Eldr, Gm30948, and Tspo2 in the three experimental groups exposed to noise on days 1, 14, and 28 compared with those in the control group (Figure 6A–E). Conversely, the analysis revealed no significant difference in the expression of Cd5l between the control group and the experimental groups (Figure 6F). This finding indicates pronounced and sustained suppression of the transcriptional processes Gm37065, F930017D23Rik, Eldr, Gm30948 and Tspo2, with a consistent pattern of downregulation observed in response to noise exposure. The specificity and persistence of these biomarkers in response to environmental noise stressors were further emphasized.

## 4 Discussion

Noise‐induced hearing loss (NIHL) refers to a type of sensorineural hearing loss caused by prolonged or intense noise exposure. It is characterized by its cumulative and irreversible nature. At present, the mechanisms underlying NIHL are not fully understood and require further investigation. Integrative transcriptomic and proteomic analyses offer significant advantages in biological research by providing comprehensive, in‐depth, and accurate information on the regulation of gene expression [23]. This helps to unravel complex biological processes and disease mechanisms. In this study, we performed a combined transcriptomic and proteomic analysis of cochlear samples collected from animals to identify biomarkers affecting noise‐induced hearing loss (NIHL). Further bioinformatic analysis of these biomarkers aims to provide effective preventive and therapeutic strategies for patients with NIHL.

On the basis of the current literature and our analysis results, six biomarkers (Gm37065, Cd5l, F930017D23Rik, Eldr, Gm30948, and Tspo2) associated with NIHL were identified. Gm37065 is a predicted gene widely expressed in the adult thymus, liver, and house mouse, but its function remains unknown [24]. Similarly, Gm30948 is a predicted gene with unknown function [25]. Their inclusion in the biomarker panel was primarily based on strong correlations with transcriptional changes associated with NIHL, but lacked functional validation (e.g., gene knockout models or protein interaction experiments). This limitation may impair the direct interpretation of their specific mechanistic roles in NIHL. Cd5l is a 40‐kDa soluble glycoprotein belonging to the scavenger receptor cysteine‐rich superfamily. This protein is widely expressed and regulates macrophage functions, such as preventing apoptosis in macrophages and other cell types, thereby participating in inflammatory processes [26]. It also activates TLRs through increased autophagy mechanisms, enhancing macrophage activity [27]. Therefore, Cd5l plays crucial roles in inflammation, tumors, and atherosclerosis. F930017D23Rik encodes a lamin A pseudogene, which does not produce a functional protein but acts as a pseudogene. Lamin A is a major component of the nuclear lamina and is crucial for maintaining the integrity of the nuclear membrane and the structure of the cell nucleus [25].

ELDR is a long noncoding RNA located downstream of EGFR in both the nucleus and the cytoplasm. Research suggests that ELDRs play important roles in mouse brain development and neural differentiation [28]. It is highly expressed in mouse and head and neck cancer samples. The lncRNA ELDR is specifically expressed in NSCs derived from embryonic stem cells, and its expression decreases during neural differentiation, suggesting its importance in maintaining the self‐renewal of neural stem cells [29]. Additionally, ELDR interacts with the RNA‐binding protein HuR in NSCs, and neither ELDR nor HuR shows significant changes in expression during the induction of NSCs from pluripotent stem cells, suggesting its importance in maintaining the pluripotent differentiation function of stem cells [30].

Tspo (translocator protein), a mitochondrial transporter and high‐affinity cholesterol‐binding protein localized to the mitochondrial outer membrane, interacts with mitochondrial permeability transition pore (mPTP) components and participates in cholesterol transport [31]. It is also involved in diverse cellular processes, including apoptosis and proliferation. Composed of 169 amino acids with five transmembrane domains, Tspo is widely expressed in steroidogenic organs and brain tissues, highly evolutionarily conserved, and its genetic deletion in mice results in embryonic lethality [32]. Early studies proposed that Tspo mediates intramitochondrial cholesterol transport to promote neurosteroidogenesis [33]. However, recent genetic knockout models challenge its essential role in this process.

Emerging evidence highlights the involvement of Tspo in mitochondrial energy metabolism. Tspo knockdown impairs cellular respiration and ATP production and modulates the balance between oxidative phosphorylation and glycolysis, favoring glycolytic flux—a mechanism implicated in glioblastoma progression [34]. Ligand binding to Tspo regulates reactive oxygen species (ROS) levels, whereas Tspo transfection enhances cellular tolerance and mitigates oxidative damage [35]. Notably, Tspo overexpression suppresses VDAC1 expression, whereas VDAC1 knockdown reciprocally reduces Tspo levels [36]. This reciprocal regulation may influence mitophagy activity by altering the expression of mitophagy‐associated proteins (e.g., Parkin and P62/SQSTM1), thereby impacting cellular fate.

Furthermore, Tspo overexpression‐induced mitophagy deficiency amplifies NLRP3 inflammasome activity, triggering inflammatory cytokine release [37]. Conversely, Tspo ligands inhibit VDAC1‐mediated proinflammatory signaling, ameliorate inflammatory responses, and restore mitophagy activity [38]. These findings underscore the multifaceted roles of Tspo in mitochondrial homeostasis, metabolic reprogramming, and inflammatory regulation, with implications for both physiological and pathological contexts.

The mechanism of action of these six biomarkers in NIHL has not yet been reported. This is the first report of a correlation between these genes and NIHL, and their mechanisms in other diseases may be similar to those in NIHL. Our results revealed that the six biomarkers were significantly enriched in chemical osmotic synthesis and uncoupling of ATP and the respiratory electron transport chain when Day 1 and the CN (control) were compared. Day 14 and CN were significantly enriched in the initial triggering of complement and the role of phospholipids in phagocytic reactions. Day 28 and CN were significantly enriched in the role of phospholipids in phagocytic reactions. These findings suggest that the development and progression of NIHL are closely related to mitochondrial energy disorders and phagocytic responses in the cell cytoplasm.

This study analyzed the molecular regulatory network of biomarkers, identifying Ctcf, Myb, and Mafb as key transcription factors. Specifically, Ctcf and Myb were found to regulate Tspo2 expression, whereas Mafb modulates Cd5l expression. Ctcf can bind chromatin to regulate multigene transcription [39], and MYB participates in cell cycle progression and proliferation [40]; however, its mechanism in Tspo2 regulation remains uncharacterized. Mafb interacts with Cd5l to mediate macrophage‐associated responses. The regulatory roles of these transcription factors in noise‐induced hearing loss (NIHL) have not been previously reported, highlighting their potential importance in elucidating disease pathogenesis.

Compared with that of the controls, RT–qPCR validation revealed significantly decreased mRNA expression of five genes (including Gm37065) across the three experimental groups, whereas Cd5l expression was not significantly different. Functional analysis suggested that mitochondrial energy metabolism dysfunction and cochlear cell inflammation may play pivotal roles in NIHL. Notably, Tspo‐regulated cholesterol metabolism might represent a critical pathological process in NIHL, although the associations between Tspo and NIHL mechanisms remain unexplored in the literature. This investigation provides novel insights into the molecular mechanisms underlying NIHL, identifying promising directions for future research on therapeutic targets and pathogenic pathways.

In conclusion, this study identified six biomarkers (Gm37065, Cd5l, F930017D23Rik, Eldr, Gm30948, and Tspo2) through a combined proteomic and transcriptomic analysis that demonstrated a strong correlation with NIHL. These biomarkers may serve as potential targets for the diagnosis and treatment of NIHL. The combined transcriptomic and proteomic approach enabled the identification of consensus biomarkers (e.g., Tspo2) that were validated across independent molecular layers, significantly strengthening the reliability of our findings. Traditional single‐omics studies often miss such integrated signals. By correlating DEPs and DEGs, we reduced false positives and highlighted molecules with functional coherence (e.g., coordinated dysregulation in NIHL progression) and characterizing their temporal correlation patterns. Furthermore, the identified molecular targets have not been previously reported in NIHL research, thereby providing novel insights to address the existing knowledge gap in this field. However, this study has certain limitations: first, he absence of objective auditory threshold data and ABR testing in SD rats limited direct quantification of noise‐induced hearing loss severity, second, the regulatory mechanisms involving the six key molecules lack in‐depth elucidation; third, further mechanistic validation and confirmation of their expression levels are necessary. Future research will focus on the detailed validation and functional intervention of the identified molecules and their associated pathways. For example, in‐depth mechanistic experiments will be conducted on molecules with established research foundations, such as Tspo and Cd5l. Regarding the predicted genes (e.g., Gm37065, Gm30948), although their direct functions remain unclear, their consistent and specific expression patterns in NIHL (e.g., sustained downregulation across multiple time points) suggest potential as biomarkers for early diagnosis or prognosis. Further exploration of their interacting partners (e.g., via co‐expression networks or proteomics) may uncover indirect functional links to NIHL pathogenesis. Notably, protein‐level and vitro validation (e.g., Western blot or immunohistochemistry, in vitro validation of TSPO and CD5L in mitochondrial energy metabolism and inflammatory responses, and in vivo assessment in a NIHL animal model) will be prioritized to confirm the functional relevance of these biomarkers, addressing the current limitation of mRNA‐centric validation. These studies will explore novel mechanisms and potential therapeutic avenues for NIHL.

## Funding

The authors have nothing to report.

## Ethics Statement

Sprague–Dawley (SD) rats (age 8–9 weeks old) were obtained from the Laboratory Animal Center of Airforce Military Medical University, Xi'an Shaanxi Province, China, for the study. The experimental protocol was strictly performed by following the protocol of the Animal Care and Use Committee of the Airforce Military Medical University. And study was approved by the medical ethics of the first affiliated hospital of the Airforce medical university (Approval NO. SYXK2024‐003).

## Consent

This manuscript has not been published in whole or in part and is not being considered for publication elsewhere. This manuscript does not violate or infringe upon any existing copyright/s license/s from any third party. All authors agreed to the publication of the manuscript.

## Conflicts of Interest

The authors declare no conflicts of interest.

## Supporting information


**Figure S1:** Volcano and heatmap of all differentially expressed genes between different groups. A and B represent the volcano and heatmap of differential genes between Day 1 versus CN; C and D represent the volcano and heatmap of differential genes between Day 14 versus CN; E and F represent the volcano and heatmap of differential genes between Day 28 versus CN. Different colored dots represent differentially expressed genes with increased/decreased/no statistical significance.
**Figure S2:** Pathway enrichment analysis of differential proteins between different groups based on KEGG database. A and B represent the differential protein KEGG database pathway enrichment analysis circle and block diagrams of Day 1 versus CN; C and D represent the enrichment analysis circle and block diagrams of differential protein KEGG database pathways between Day 14 versus CN; E and F represent the enrichment analysis circle and block diagrams of differential protein KEGG database pathways between Day 28 versus CN. The lines of different colors in the circle diagram represent different pathways. The different colors in the block diagram represent different *p*‐values, and the larger the block area of the pathway, the more proteins are enriched.
**Figure S3:** Pathway enrichment analysis of differentially expressed genes between different groups based on the Go database. A,B represent the enrichment analysis circle and block diagram of the differential gene go database pathway between Day 1 versus CN; C,D represent the differential genes of Day 14 versus CN in the GO database pathway enrichment analysis circle diagram and block diagram; E,F represent the enrichment analysis circle and block diagrams of differential genes in the go database for Day 28 versus CN. The different colors in the circle chart represent different types of pathways, and the different circles represent the number of relative genes in the enriched pathways. Different colors in the block diagram represent different *p*‐values, and the larger the block area of the pathway, the more genes are enriched.
**Figure S4:** Pathway enrichment analysis of differentially expressed genes between different groups based on the KEGG database. A and B represent the KEGG database pathway enrichment analysis circle and block diagrams of differential genes between Day 1 versus CN; C and D represent the KEGG database pathway enrichment analysis circle and block diagrams of differential genes between Day 14 versus CN; E and F represent the KEGG database pathway enrichment analysis circle and block diagrams of differential genes between Day 28 versus CN. The lines of different colors in the circle diagram represent different pathways. Different colors in the block diagram represent different *p*‐values, and the larger the block area of the pathway, the more genes are enriched.
**Figure S5:** GSEA analysis of key genes Day 14 versus CN. A, B, C, D, E, and F represent the GSEA enrichment of Cd5l, Eldr, F930017D23Rik, Gm30948, Gm37065, and Tspo2, respectively.
**Figure S6:** GSEA analysis of key genes in Day 28 versus CN. A–F represent the GSEA enrichment of Cd5l, Eldr, F930017D23Rik, Gm30948, Gm37065, and Tspo2, respectively.
**Table S1:** Procedure of RT‐qPCR.
**Table S2:** Information on primer sequences.
**Table S3:** The concentration of total RNA.
**Table S4:** Key Biomarker Selection Criteria and Cross‐Omics Validation.

## Data Availability

The data that support the findings of this study are available on request from the corresponding author. The data are not publicly available due to privacy or ethical restrictions.
