# Supplementary material for: Application of a Combined Transcriptomics and Proteomics Analysis Approach to Explore Potential Mechanisms of Biomarkers in NIHL
Source: CNS Neurosci Ther. 2026 Aug 27;32(8):e70935. doi: 10.1002/cns.70935 (PMC13520924; doi:10.1002/cns.70935)
Supplement: Supplementary file 1 [file CNS-32-e70935-s001.zip › cns70935-sup-0007-TableS1.docx]

**Supplementary Table 1 Procedure of RT-qPCR**

|  | **temperature** | **time** |
| --- | --- | --- |
| Pre-denaturation | 95℃ | 1min |
| denaturation | 95℃ | 20s |
| anneal | 55℃ | 20s |
| extend | 72℃ | 30s |
