# Supplementary material for: Application of a Combined Transcriptomics and Proteomics Analysis Approach to Explore Potential Mechanisms of Biomarkers in NIHL
Source: CNS Neurosci Ther. 2026 Aug 27;32(8):e70935. doi: 10.1002/cns.70935 (PMC13520924; doi:10.1002/cns.70935)
Supplement: Supplementary file 1 [file CNS-32-e70935-s001.zip › cns70935-sup-0008-TableS2.docx]

**Supplementary Table 2 Information on primer sequences**

| **primers** | **sequences** | |
| --- | --- | --- |
| Gm37065 F | TTGAGGCAATGTAAGGGCGA | |
| Gm37065 R | TTCCCGGAGGCTCTTCTAGT | |
| Cd5l F | GCGAGGACACATGGATGGAA |  |
| Cd5l R | GCCCCAGGCCCATAGATTTT |  |
| F930017D23Rik F | GCAAAAGATGCCGAGAACCC |  |
| F930017D23Rik R | GACCACCTGCTTTTGTTGGC |  |
| Eldr F | GCCAAGGGAGATTGTGGAGA |  |
| Eldr R | CACAATCCTCAGGTGGCAGG |  |
| Gm30948 F | TGAAGGTGAGCAGCCATGAAG |  |
| Gm30948 R | CCTCTAGCACTCTGGCTCTTG |  |
| Tspo2 F | GACCAACCTGGATCTTGCCT |  |
| Tspo2 R | CTCATCCTCACACCGACTGG |  |
| GAPDH F | GACCCCTTCATTGACCTCAAC |  |
| GAPDH R | GCCATCACGCCACAGCTTTCC |  |
