# Supplementary material for: Application of a Combined Transcriptomics and Proteomics Analysis Approach to Explore Potential Mechanisms of Biomarkers in NIHL
Source: CNS Neurosci Ther. 2026 Aug 27;32(8):e70935. doi: 10.1002/cns.70935 (PMC13520924; doi:10.1002/cns.70935)
Supplement: Supplementary file 1 [file CNS-32-e70935-s001.zip › cns70935-sup-0009-TableS3.docx]

**Supplementary Table 3 The concentration of total RNA**

| **Number** | **Group** | **Concentration**  **(ng/uL)** | **A260/A280** | **A260/A230** |
| --- | --- | --- | --- | --- |
| 1 | Control | 119.40 | 1.61 | 0.437 |
| 2 | Control | 79.16 | 1.484 | 0.089 |
| 3 | Control | 191.32 | 1.45 | 0.362 |
| 4 | D1 | 101.60 | 1.348 | 0.253 |
| 5 | D1 | 139.00 | 1.589 | 0.504 |
| 6 | D1 | 125.84 | 1.547 | 0.425 |
| 7 | D14 | 129.28 | 1.406 | 0.359 |
| 8 | D14 | 202.48 | 1.476 | 0.41 |
| 9 | D14 | 150.52 | 1.497 | 0.357 |
| 10 | D28 | 156.40 | 1.595 | 0.538 |
| 11 | D28 | 94.72 | 1.5367 | 0.361 |
| 12 | D28 | 106.72 | 1.490 | 0.274 |
