# Supplementary material for: Application of a Combined Transcriptomics and Proteomics Analysis Approach to Explore Potential Mechanisms of Biomarkers in NIHL
Source: CNS Neurosci Ther. 2026 Aug 27;32(8):e70935. doi: 10.1002/cns.70935 (PMC13520924; doi:10.1002/cns.70935)
Supplement: Supplementary file 1 [file CNS-32-e70935-s001.zip › cns70935-sup-0010-TableS4.docx]

| **Gene** | **DEG1_log2FC** | **DEG1_padj** | **DEG2_log2FC** | **DEG2_padj** | **DEG3_log2FC** | **DEG3_padj** |
| --- | --- | --- | --- | --- | --- | --- |
| Gm37065 | -2.034859477 | 0.00217206761168839 | -6.138134295 | 1.39806558903286e-06 | -4.112727978 | 4.76415546722422e-08 |
| Cd5l | -1.502513797 | 0.00154809152463777 | -2.848436533 | 3.6828128681149e-09 | -2.843521765 | 5.77925279158718e-09 |
| F930017D23Rik | -1.459965914 | 0.000777826725779755 | -2.098409486 | 3.08228521143026e-05 | -2.151899689 | 1.81107754221782e-05 |
| Eldr | -1.006531087 | 0.0345268474166544 | -1.181990317 | 0.0155539962597033 | -1.047430254 | 0.00264964935399927 |
| Gm30948 | -1.771256661 | 9.41197502979977e-05 | -4.401335828 | 2.08712103105965e-11 | -3.863097849 | 2.89005776880199e-12 |
| Tspo2 | -1.460845029 | 0.000385563169968076 | -2.794170688 | 1.44557754972342e-08 | -2.583718301 | 2.09738353720475e-18 |

**Supplementary Table 4 Key Biomarker Selection Criteria and Cross-Omics Validation**
